# First the B cells fall, then the T cells follow: temporal immunological shift with ocrelizumab in multiple sclerosis

**DOI:** 10.1007/s00415-025-13297-5

**Published:** 2025-08-16

**Authors:** Gianmarco Abbadessa, Elisabetta Maida, Simona Bonavita, Luigi Lavorgna

**Affiliations:** 1 Department of Advanced Medical and Surgical Sciences, University of Campania Luigi Vanvitelli, Via Pansini 5, 80131 Naples, Italy; 2 Department of Brain Sciences, Imperial College London, London, W120BZ UK

**Keywords:** Multiple sclerosis, Ocrelizumab, B cells, T cells, Gene set variation analysis

## Abstract

**Background:**

Ocrelizumab (OCR) is a humanized anti-CD20 monoclonal antibody approved for multiple sclerosis (MS). While its efficacy has been attributed to early and sustained B cell depletion, emerging evidence suggests a broader immunomodulatory profile.

**Objectives:**

To investigate temporal dynamics of OCR-induced immune modulation in MS by analyzing pathway enrichment score changes in transcriptomic data from peripheral blood mononuclear cells (PBMCs) at early (2 weeks) and late (6 months) timepoints following treatment initiation.

**Methods:**

We analyzed publicly available microarray data (GSE228330) from PBMCs of 15 MS patients treated with OCR. Immune cell subpopulations were estimated using CIBERSORTx with the LM22 signature matrix. Gene Set Variation Analysis (GSVA) was applied to quantify immune-related pathway enrichment across three timepoints—baseline, 2 weeks, and 6 months.

**Results:**

Early effects were characterized by selective suppression of B cell-related pathways, including antigen presentation via MHC-II, B cell proliferation, and survival. These changes were accompanied by compensatory upregulation of anti-inflammatory and innate immune signaling (e.g., IL-10, monocyte chemotaxis). At 6 months, B cell pathway suppression persisted and deepened, while T cell-specific pathways (e.g., CD4+ T-cell activation and cytokine production) showed significant downregulation, indicating a delayed but substantial impact on adaptive cellular immunity. At 6 months, T reg compartment was reconfigured, with overall T reg transcription enhanced versus T effector cells, quiescent and thymic‑mature signatures reduced, and IL‑4-induced T reg program enriched.

**Conclusions:**

OCR exerts a biphasic immunomodulatory effect, with rapid direct suppression of B cell pathways followed by delayed indirect modulation of T cell-mediated immunity.

**Supplementary Information:**

The online version contains supplementary material available at 10.1007/s00415-025-13297-5.

## Introduction

Ocrelizumab (OCR) is an intravenously administered, humanized monoclonal antibody targeting the CD20 antigen expressed on the surface of immature and mature circulating B cells, leading to their depletion [1]. This effect is observed within the first few weeks after treatment administration and represents the primary immunological mechanism of action. Over time, additional delayed effects also emerge, including alterations on B cell repertoire repopulating the peripheral compartment, with a predominance of naïve B cells and a sustained reduction in memory B cells [2]. In addition, B cell depletion triggers a cascade of downstream effects that also influence T cell function, notably by reducing the antigen-presenting capacity of B cells, thereby modulating the activity of autoreactive T cells [3–5]. Moreover, a decline in the expression of activation and migration markers is observed on CD8^+^ T cells and Natural Killer (NK) cells, along with a reduction in their in vitro cytotoxic activity [6].

Thanks to these mechanisms, OCR has emerged as a major therapeutic advancement in the management of Multiple Sclerosis (MS). It has demonstrated significant efficacy in both relapsing MS (RMS) and primary progressive MS (PPMS), effectively reducing disease activity and slowing progression [7, 8]. The long-term clinical benefits of OCR have been confirmed by data from the OLE phases, where patients monitored for up to 7.5 years experienced sustained reductions in disability progression and a lower likelihood of requiring walking assistance [9–11].

The aim of this study was to characterize the timing of OCR’s immunological effects using GSVA to analyze transcriptomic changes in peripheral blood mononuclear cells (PBMCs), assessing immune-related pathway enrichment changes at early (2–4 weeks) and late (6 months) timepoints after treatment.

## Methods

### Data source and study design

We used publicly available transcriptome data (GEO accession: GSE228330) generated from PBMCs of MS patients undergoing OCR therapy. The cohort comprised 15 OCR–MS treated individuals. Samples were collected at three timepoints: pre-therapy (baseline), 2 week post-infusion, and 6-month post-infusion. The gene expression profiling was performed using the Affymetrix Human Clariom D Assay, which interrogates over 135,000 transcripts, thus providing a comprehensive landscape of gene activity [12].

### Preprocessing and normalization

Raw microarray data were processed to generate transcripts per million (TPM) values, which normalized gene expression levels across samples, ensuring comparability. Rigorous quality control steps were applied during the normalization process to minimize technical variability and to prepare the data for downstream enrichment analysis.

### Gene mapping

The probeset IDs were mapped to gene symbols using the clariomdhumantranscriptcluster.db database, with rows updated to reflect the corresponding gene symbols. Subsequently, rows with missing gene symbols were removed and duplicate entries were collapsed by taking their mean expression values.

### CIBERSORTx deconvolution

The gene expression matrix obtained from GEO was first subjected to immune cell deconvolution using the CIBERSORT algorithm [13], employing the LM22 signature matrix. CIBERSORT is a computational method that leverages gene expression profiles to estimate the relative proportions of distinct immune cell subtypes within a mixed cell population. The LM22 signature matrix, which comprises 547 genes, is specifically curated to distinguish 22 human hematopoietic cell phenotypes, including various B cells, T cells, NK cells, macrophages, dendritic cells, and other immune subsets. This matrix has been extensively validated and is widely used to characterize the immune landscape in tissue samples, thereby providing critical insights into the immune contexture of complex diseases.

Following deconvolution, we performed statistical analyses to compare the estimated immune cell fractions across different treatment durations. Specifically, the Kruskal–Wallis test was applied to assess differences among treatment groups for each immune cell subtype, with subsequent Dunn post-hoc tests (using Bonferroni correction) for pairwise comparisons where significant differences were observed.

### Gene set variation analysis

To quantify the enrichment of predefined biological pathways in each sample, we applied gene set variation analysis (GSVA), a non-parametric, unsupervised method that transforms gene expression data into pathway enrichment scores, capturing subtle pathway activity differences without the need for predetermined class labels. We queried msigdbr to assemble a comprehensive panel of gene sets—Gene Ontology (GO) Biological Process, Molecular Function, Cellular Component (C5), Reactome, PID, Wikipathwys and Biocarta curated pathways (C2), Hallmarks (H), and Immunologic Signatures (C7). The TPM-normalized expression matrix was then subjected to GSVA using a Gaussian kernel to accommodate the continuous nature of the data, focusing only on gene sets containing between 15 and 500 genes to ensure each pathway was robustly represented; the resulting GSVA scores, reflecting the relative enrichment of each pathway in each sample, were visualized using heatmaps to assess global activity patterns. To identify pathways significantly modulated by OCR therapy, we compared GSVA scores across three groups—baseline (pre-therapy), 2 weeks, and 6-month post-therapy—using the Shapiro–Wilk test to assess normality (adopting a conservative non-parametric approach for groups with fewer than three samples) and an *F* test to evaluate variance homogeneity; depending on these results, either a parametric *t* test or a non-parametric Wilcoxon rank-sum test was applied, with comparisons made between baseline and 2 weeks (0 vs. 0.5) as well as between baseline and 6 months (0 vs. 6). Pathways that demonstrated a *p* value <0.05 in both comparisons were considered significantly modulated, with those meeting this threshold at both timepoints representing early effects and those significant only at the 6-month follow-up indicating late effects.

## Results

### Ocrelizumab selectively depletes B cell subsets

Our analysis revealed that for B cell subtypes, including naive, memory, and plasma cells, there were significant differences across treatment durations (Table 1), and these were evident already 2 weeks after the first administration. In contrast, no significant differences were observed for T cell subsets (such as CD8, CD4 naïve, CD4 memory resting, CD4 memory activated, regulatory T cells (T regs), and gamma–delta T cells) as well as for NK cells, monocytes, and other myeloid populations (Table 1).

**Table 1:** Comparison of immune cell proportions across three timepoints—baseline (0), 2 weeks (0.5), and 6 months (6)—as estimated by deconvolution using CIBERSORTx

Cell subtype	KW chi-squared (df)	KW *p* value	Dunn test comparisons (Bonferroni adjusted *p* values and Z-scores)	Comments
B cells naive	18.37 (2)	0.0001026	0 vs 0.5: *Z* = 4.1904, *p* = 8.35e-05; 0 vs 6: *Z* = 2.8430, *p* = 0.01341; 0.5 vs 6: *Z* = −1.3968, *p* = 0.4874	Significant differences
B cells memory	12.89 (2)	0.001586	0 vs 0.5: Z = 2.4637, *p* = 0.04125; 0 vs 6: *Z* = 3.4856, *p* = 0.00147; 0.5 vs 6: *Z* = 0.9612, *p* = 1.0000	Significant differences
Plasma cells	16.54 (2)	0.0002565	0 vs 0.5: *Z* = −3.7005, *p* = 0.00065; 0 vs 6: *Z* = −3.2867, *p* = 0.00304; 0.5 vs 6: *Z* = 0.4710, *p* = 1.0000	Significant differences
T cells CD8	0.61365 (2)	0.7358	–	No significant differences
T cells CD4 naive	1.4599 (2)	0.4819	–	No significant differences
T cells CD4 memory resting	0.72975 (2)	0.6943	–	No significant differences
T cells CD4 memory activated	3.3705 (2)	0.1854	–	No significant differences
T cells follicular helper	–	–	–	Univocal values; test skipped
T cells regulatory (Tregs)	2.1687 (2)	0.3381	–	No significant differences
T cells gamma delta	2.1429 (2)	0.3425	–	No significant differences
NK cells resting	2.0637 (2)	0.3564	–	No significant differences
NK cells activated	1.2251 (2)	0.5420	–	No significant differences
Monocytes	0.57071 (2)	0.7517	–	No significant differences
Macrophages M0	–	–	–	Univocal values; test skipped
Macrophages M1	2.1429 (2)	0.3425	–	No significant differences
Macrophages M2	3.4072 (2)	0.1820	–	No significant differences
Dendritic cells resting	–	–	–	Univocal values; test skipped
Dendritic cells activated	3.2227 (2)	0.1996	–	No significant differences
Mast cells resting	1.059 (2)	0.5889	–	No significant differences
Mast cells activated	0.83299 (2)	0.6594	–	No significant differences
Eosinophils	2.0633 (2)	0.3564	–	No significant differences
Neutrophils	2.9918 (2)	0.2240	–	No significant differences

For each immune cell subtype, a Kruskal–Wallis (KW) test was first performed to assess overall differences among the timepoints. The KW test results, including the chi-squared statistic with its corresponding degrees of freedom (df) and *p* value, are reported in the second and third columns

Subsequently, pairwise comparisons were conducted using Dunn’s test with Bonferroni adjustment for multiple comparisons. The Dunn test results include Z-scores and the Bonferroni-adjusted *p* values for the following comparisons:

• Baseline vs 2 weeks (0 vs 0.5)

• Baseline vs 6 months (0 vs 6)

• 2 weeks vs 6 months (0.5 vs 6)

For each cell subtype, the “Comments” column summarizes whether the differences were statistically significant. For some cell types with identical (univocal) values across the timepoints, statistical testing was not applicable (“test skipped”)

### Ocrelizumab modulation of immune-related pathway

#### Early effects: B cell-mediated antigen presentation suppression

The pathway for B cell proliferation dropped by approximately 0.16 (from +0.139 to −0.018; *p* = 0.0027) at 2 weeks and by about 0.20 (from +0.139 to −0.064; *p* = 0.0021) at 6 months. In addition, we observed a coordinated remodeling of B cell apoptotic signatures: the core apoptotic process dropped by approximately 0.17 (from +0.121 to −0.044; *p* = 0.032) at 2 weeks and by about 0.18 (from +0.121 to −0.059; *p* = 0.042) at 6 months; the general regulation of apoptosis fell by roughly 0.19 (from +0.160 to −0.032; *p* = 0.018) at 2 weeks and by about 0.22 (from +0.160 to −0.061; *p* = 0.014) at 6 months; conversely, the negative regulation of extrinsic apoptotic signaling pathway rose by approximately 0.12 (from −0.080 to +0.038; *p* = 0.031) at 2 weeks and by about 0.13 (from −0.080 to +0.046; *p* = 0.049) at 6 months.

At 2 weeks (*p* = 0.0459) and 6 months (*p* = 0.0408) post-treatment, we observed a pronounced downregulation of pathway activity related to antigen processing and presentation via Major Histocompatibility Complex (MHC) class II molecules compared to baseline. Moreover, the MHC class II protein complex decreased by about 0.45 (from +0.313 to −0.140; *p* = 0.0023) and the broader MHC protein complex by roughly 0.30 (from +0.208 to −0.090; *p* = 0.0223), reinforcing the impaired functionality of antigen presentation. Likewise, the assembly of the peptide antigen–MHC protein complex, essential for T cell activation, decreased by nearly 0.39 (from +0.285 to −0.102; *p* = 0.0027) at 2 weeks and by roughly 0.43 (from +0.285 to −0.143; *p* = 0.0164) at 6 months.

The pathway corresponding to the positive regulation of monocyte chemotaxis increased by approximately 0.29 (from −0.180 to +0.108; *p* = 0.0022) at 2 weeks and by around 0.26 (from −0.180 to +0.076; *p* = 0.0143) at 6 months.

Complementary insights were obtained from curated pathway analyses. In BIOCARTA, the B lymphocyte pathway decreased by approximately 0.08 (from −0.408 to −0.490; *p* = 0.0445), mirroring the direct effects of B cell depletion, while the IL-10 pathway increased by about 0.16 (from −0.314 to −0.154; *p* = 0.0422) suggesting a potential rise in anti-inflammatory signaling despite suppressed adaptive immunity. From the PID database, several cytokine and immune regulatory pathways were significantly upregulated: the IL-3 (multi-colony stimulating factor; multi-CSF) pathway increased by roughly 0.23 (from −0.148 to +0.086; *p* = 0.0041), the lymph angiogenesis pathway by about 0.21 (from −0.168 to +0.043; *p* = 0.0185), and the T cell protein tyrosine phosphatase (TCPTP) pathway by nearly 0.26 (from −0.156 to +0.108; *p* = 0.0051). All pathway enrichment scores, and corresponding statistical comparisons are reported in Supplementary Tables S1–S9.

Overall, the effects already evident after 2 weeks are almost exclusively related to B cell depletion—manifesting as significant decreases in antigen presentation, B cell proliferation, and apoptotic regulation—accompanied by immune compensatory mechanism, mainly related to the innate immune system.

#### Late effects: T cell activity suppression

At 6-month post-anti‐CD20 therapy, the suppression of B cell functions that was evident early on is maintained and, in several cases, becomes even more pronounced. First, the B cell differentiation pathway, which had a baseline mean expression of 0.0965, declines to −0.0392 at 6 months (*p* = 0.0323). In addition to the early changes observed in some B cell apoptosis-related pathways, at 6 months we also detected a pronounced downshift in the negative regulation of B cell apoptotic process signature, from +0.162 at baseline to −0.1145 at 6 months (*p* = 0.0039). In parallel, markers associated with the positive regulation of B cell proliferation continue to decline, reflecting a persistent reduction in the capacity for B cell expansion. These robust changes in B cell-associated pathways confirm that anti‐CD20 therapy exerts a long‐lasting and direct suppressive effect on the B cell compartment.

Of note, several T cell-related pathways also exhibit significant late alterations that were not as evident at the early timepoint. The pathway for CD4+, αβ T cell cytokine production, which was initially measured at a baseline mean of 0.1346, decreases markedly to −0.1185 at 6 months (*p* = 0.0329). In addition, the positive regulation of CD4+, αβ T cell activation is significantly diminished, dropping from 0.1178 at baseline to −0.0745 at 6 months (*p* = 0.0284). Finally, regulatory T cell differentiation pathways showed a significant decline from 0.1016 at baseline to −0.1322 at 6 months (*p* = 0.0032).

Further dissection using IMMUNESIGDB gene sets (Supplementary Table S15) provided a more nuanced view of T reg remodeling at 6 months. The “resting” T reg signature declined modestly from 0.0410 at baseline to −0.0098 (*p* = 0.0481), indicating a mild loss of quiescent, homeostatic T regs. Similarly, the mature thymic T reg program showed a significant decrease, from 0.0679 to −0.0891 (*p* = 0.0458), suggesting that thymic maturation of T regs was compromised 6 months after OCR administration. In contrast, the IL‑4-induced conversion T reg signature increased, as indicated by the GSE24634_NAIVE_CD4_TCELL_VS_DAY7_IL4_CONV_TREG_UP score decreasing from 0.1278 to −0.0483 (*p* = 0.0235), suggesting potentiated peripheral generation of new T regs. Finally, the T reg versus T effector signature was enriched, rising from −0.0376 to +0.0383 (*p* = 0.0235), consistent with the preservation—or even potentiation—of the core regulatory transcriptional program relative to effector T cells.

Turning to the innate immune system, the late effects revealed a somewhat different pattern that emerged only at 6 months. One notable observation is the significant increase in the regulation of leukocyte tethering or rolling, which rises from −0.1139 at baseline to 0.0909 at 6 months (*p* = 0.0404). Other innate immune markers, such as those associated with the negative regulation of viral entry into host cells, also show significant alterations—for instance, a reduction from 0.1634 at baseline to −0.1266 at 6 months (*p* = 0.0135). A comprehensive overview of these late-stage pathway alterations, including T cell and innate immune responses, is presented in Supplementary Tables S10–S18.

**Fig. 1:**
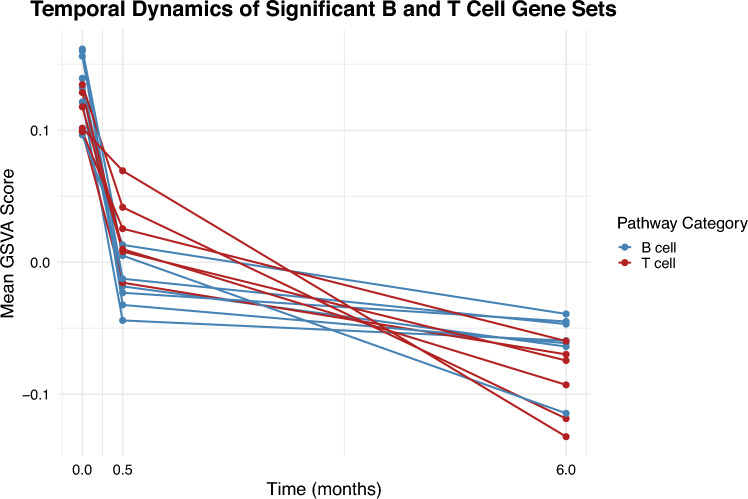
Temporal trends of mean expression values at timepoints 0, 0.5, and 6 for GO biological process selected pathways. It is evident that the effect on B cell-related pathways is both immediate and sustained, whereas the impact on T cell-related pathways develops more gradually, with a significantly more pronounced effect observed at 6 months compared to 2 weeks. T cell-related pathways: GOBP_NEGATIVE_REGULATION_OF_T_CELL_DIFFERENTIATION, GOBP_CD4_POSITIVE_ALPHA_BETA_T_CELL_CYTOKINE_PRODUCTION, GOBP_POSITIVE_REGULATION_OF_CD4_POSITIVE_ALPHA_BETA_T_CELL_ACTIVATION, GOBP_POSITIVE_REGULATION_OF_REGULATORY_T_CELL_DIFFERENTIATION, GOBP_REGULATION_OF_CD4_POSITIVE_ALPHA_BETA_T_CELL_ACTIVATION, and GOBP_REGULATORY_T_CELL_DIFFERENTIATION. B cell-related pathways GOBP_B_CELL_APOPTOTIC_PROCESS, GOBP_B_CELL_PROLIFERATION, GOBP_POSITIVE_REGULATION_OF_B_CELL_PROLIFERATION, GOBP_REGULATION_OF_B_CELL_APOPTOTIC_PROCESS, GOBP_REGULATION_OF_B_CELL_PROLIFERATION, GOBP_B_CELL_DIFFERENTIATION, and GOBP_NEGATIVE_REGULATION_OF_B_CELL_APOPTOTIC_PROCESS

Figure 1 displays the enrichment scores over time for Gene Ontology biological processes related to B cells and T cells. It is evident that the effect on B cell-related pathways is both immediate and sustained, whereas the impact on T cell-related pathways develops more gradually, with a significantly more pronounced effect observed at 6 months compared to 2 weeks.

In summary, the late effects of anti‐CD20 therapy at 6 months were characterized by a persistent and robust suppression of B cell-specific pathways, accompanied by significant downregulation of key T cell-mediated adaptive responses that were either maintained from the early phase or emerged only at 6 months. Notably, the T reg compartment underwent a shift from quiescent and thymic‑derived populations toward activated, IL‑4-induced and effector‑like phenotypes, suggesting compensatory maintenance—and even potentiation—of regulatory function. These findings underscore the enduring immunomodulatory impact of anti‐CD20 therapy in MS, with important implications for both its therapeutic efficacy and the long-term maintenance of immune homeostasis.

## Discussion

The present study aimed to characterize the temporal dynamics of OCR-induced immunological changes by applying GSVA to transcriptomic data derived from PBMCs at early (2–4 weeks) and late (6 months) timepoints. Our findings highlight the early and selective depletion of B cell subsets, accompanied by distinct transcriptional remodeling in immune-related pathways. These alterations evolve over time, leading to a delayed impact on T cell-mediated adaptive responses while leaving most innate immune components relatively unaffected. Understanding this sequential pattern may be critical to optimizing the timing and duration of anti-CD20 therapy in MS.

First, early in the treatment course, OCR exerts a rapid and selective effect on the B cell compartment [14, 15]. This was evidenced by the marked depletion of circulating CD20+ B cell and the consistent downregulation of B cell-specific transcriptional programs, including those regulating antigen presentation, proliferation, and survival. Pathways such as MHC class II-mediated antigen processing, B cell apoptotic regulation, B cell proliferation, and peptide-MHC complex assembly showed significant reductions in enrichment scores within weeks of treatment. In particular, the downregulation of peptide–MHC complex formation, is highly relevant in the context of MS, where autoreactive CD4+ T cells are thought to play a central role in pathogenesis [16]. These transcriptomic changes reflected the immediate B cell depletion occurring after OCR administration. Of note, just 2 weeks after OCR treatment we observed a coordinated reduction in both core apoptotic machinery and general apoptosis‑regulation signatures, accompanied by an increase in negative regulators of the extrinsic (death‑receptor) pathway. All three of these shifts remained significant at 6 months, at which point we also detected a further reduction in the intrinsic anti‑apoptotic “brakes” specific to B cells. Because these profiles derive from bulk PBMCs, the observed shifts most likely reflect ’s selective elimination of CD20⁺ B cells via ADCC/CDC, followed by the preferential survival and transcriptional reprogramming of both the remaining CD20⁺ subset and other non‑depleted (CD20⁻) populations. These durable alterations in apoptotic pathway enrichment underscore a lasting recalibration of survival programs post‑therapy and warrant further investigation at the single‑cell and functional levels.

In contrast, no significant transcriptomic alterations were observed in T cell-related pathways during the early phase, highlighting the specificity of OCR’s initial pharmacodynamic profile. This finding aligns with previous reports showing stable CD4+ and CD8+ T cell counts and preserved functional responses in the early months after treatment, despite profound B cell depletion [17].

Interestingly, several innate immune pathways appeared upregulated at both early and late timepoints. Most notably, we observed an upregulation in the expression of genes involved in monocyte chemotaxis, possibly reflecting a compensatory recruitment of innate immune cells in response to B cell loss. In addition, the IL-10 signaling pathway was also upregulated, suggesting an early shift toward anti-inflammatory signaling. Similarly, additional increases in the IL-3 pathway, lymphangiogenesis pathway, and the TCPTP pathway highlights a broader reconfiguration of cytokine signaling and innate immune activation.

These findings are strongly supported and complemented by the recent single-cell transcriptomic study by Wei et al. [18]. Indeed, they demonstrated that B cell depletion led to increased frequencies of peripheral CD16+ monocytes and CSF-specific macrophages, with these myeloid populations exhibiting an anti-inflammatory transcriptomic signature. Notably, their study also found upregulation of anti-inflammatory genes such as IL-10 in CSF macrophages, and increased TNFα expression in peripheral blood monocytes, which they propose contributes to a therapeutic anti-inflammatory effect in MS. All together these findings provide evidence for a systemic immunological rebalancing and a shift of the innate immune compartment towards a more homeostatic or anti-inflammatory state following OCR therapy in MS.

By the 6-month post-treatment, we observed transcriptional changes extending beyond the suppression of the B cell compartment to involve several key T cell-specific pathways [6, 19]. Specifically, pathways related to CD4+ αβ T cell activation, cytokine production and regulatory T cell differentiation, show significant downregulation only at 6 months, suggesting a delayed suppression of T cell effector functions. These findings indicate that prolonged B cell depletion may eventually impair the broader adaptive immune network likely through disrupted antigen presentation, diminished co-stimulatory signals, and alterations in cytokine networks. Such delayed modulation of adaptive immunity may represent a late biological effect contributing to the long-term efficacy of OCR in MS.

In addition, we noticed that OCR induced not a simple loss but a nuanced reconfiguration of the T reg compartment. We observed significant downregulation of both the quiescent “resting” T reg signature and the mature thymic T reg signature, indicating erosion of the homeostatic reservoir and impaired thymic output. Yet, concurrently, the IL 4-induced T reg conversion and T reg versus T effector signatures were enriched, suggesting that the core suppressive machinery of remaining T regs is preserved—or even potentiated—relative to effector T cells. Drawing on Wei et al.’s single cell study, which likewise showed depletion of naïve and intermediate T regs alongside expansion of TIGIT⁺ HLA DR⁺/CD74⁺ effector T regs, we saw a coherent picture in which conventional differentiation axes are suppressed but a compensatory pathway—likely driven by CD16⁺ monocyte-derived TNFα signaling through TNFR2—stabilizes and amplifies effector like T regs [18]. Together, these data suggest that B cell depletion does not undermine regulatory capacity per se but rather reprograms the T reg pool toward a more activated, resilient phenotype, thereby sustaining long term immunomodulatory efficacy in MS.

Notably, these delayed effects on T cell transcription programs were not mirrored by significant changes in T cell proportions, as revealed by immune deconvolution, reinforcing the interpretation that OCR modulates T cell function rather than quantity. This aligns with recent reports describing phenotypic shifts in T cell subsets and a reduction in their activation and migration capacity following prolonged OCR exposure [20].

Finally, several innate immune pathways also exhibit statistically significant changes that are unique to the 6-month timepoint. We noted an upregulation of genes involved in the regulation of leukocyte tethering and rolling, a process central to immune cell trafficking which was not prominent at earlier stages. While this may represent a compensatory response to prolonged B cell depletion, it could also reflect relative changes in immune cell composition rather than a true increase in innate immune activity. Similarly, alterations in pathways related to antiviral responses may result from transcriptional rebalancing within the PBMC pool, rather than indicating enhanced antiviral defense per se. The apparent preservation, and in some cases upregulation, of innate immune functions over time may contribute to the favorable safety of OCR, maintaining basal immune competence even in the face of sustained adaptive suppression.

In this study, transcriptomic profiling of PBMCs following anti‐CD20 therapy with OCR revealed an early, robust depletion of B cell subsets accompanied by immediate downregulation of B cell-specific transcriptional programs. Notably, while these early changes reflect the rapid pharmacodynamic effects of OCR, a distinct, delayed suppression of T cell-associated pathways emerged only at the 6‐month timepoint. Although our investigation was limited to transcriptomic data and lacked direct clinical correlations, this pattern of delayed T cell modulation aligns with published clinical studies demonstrating that significant improvements in relapse rates, disability progression, and new lesion formation on magnetic resonance typically become apparent later after treatment initiation [7, 21–23]. Thus, we hypothesize that the late downregulation of T cell activation, cytokine production, and regulatory T cell differentiation observed in our data may serve as a molecular surrogate of the time period required for anti‐CD20 therapy to exert its full clinical and radiological benefits in MS. Moreover, the concurrent modulation of innate immune pathways, likely representing compensatory mechanisms to preserve immune surveillance, further underscores the complexity of the immunological rebalancing induced by OCR [24]. This reshaping of immune responses, coupled with delayed effects on T cell effector functions and preserved antiviral pathways, may underline OCR’s favorable balance between efficacy and safety [25].

This study has several limitations. First, the relatively small sample size limits the generalizability of our findings and may reduce the statistical power. Second, while GSVA provides valuable insights into pathway-level alterations, it does not capture cell-type-specific transcriptional dynamics with the resolution offered by single-cell RNA sequencing. Third, the use of bulk PBMCs inherently constrains the interpretability of tissue-specific immune mechanisms, particularly those occurring within the central nervous system, which is the primary site of pathology in MS. Finally, the lack of paired functional assays (e.g., cytokine secretion, proliferation, or antigen-specific responses) limits our ability to draw direct conclusions about the immunological consequences of the observed transcriptomic shifts.

In addition to the above, our conclusion is further constrained by the timing of available transcriptomic data. Although radiological studies such as HERMES have demonstrated early T cell-mediated effects of B cell depletion as soon as 4–6-week post-treatment, we were unable to interrogate these intermediate timepoints due to the absence of publicly accessible bulk or single-cell RNA-seq datasets at 4–8 or 12 weeks. Consequently, we cannot exclude—and indeed find it plausible—that OCR induces transcriptional changes in T cell compartments earlier than 6 months; our analysis simply lacked the temporal resolution necessary to detect such effects. This gap underscores the need for future longitudinal sampling at multiple intermediate intervals to fully map the kinetics of adaptive immune modulation following anti-CD20 therapy [26].

## Conclusion

In summary, our data indicate that anti‑CD20 treatment leads to swift B cell depletion, whereas alterations in T cell-related pathways emerge more gradually and become most noticeable by 6 months. However, we cannot rule out the possibility of earlier T cell effects—analogous to the 6–8‑week MRI improvements seen with rituximab—that our sampling intervals may have missed. Further investigation at intermediate timepoints will be necessary to clarify the true onset of these transcriptomic changes and their value as predictors of long‑term therapeutic outcomes.

## Supplementary Information

Below is the link to the electronic supplementary material.

Supplementary file1 (XLSX 52 KB)

Supplementary file2 (XLSX 56 KB)

## Data Availability

All data supporting the findings of this study are publicly available at the NCBI Gene Expression Omnibus (GEO) under accession GSE228330 (https://www.ncbi.nlm.nih.gov/geo/query/acc.cgi?acc=GSE228330). The repository includes the raw CEL files and processed expression matrices used in our analyses. No restrictions apply to the availability of these data.
